# Cooperative Regulation of Flagellar Synthesis by Two EAL-Like Proteins upon Salmonella Entry into Host Cells

**DOI:** 10.1128/spectrum.02859-22

**Published:** 2023-02-07

**Authors:** Yingying Yue, Weiwei Wang, Yue Ma, Nannan Song, Haihong Jia, Cuiling Li, Qi Wang, Hui Li, Bingqing Li

**Affiliations:** a Department of Clinical Laboratory, Shandong Provincial Hospital Affiliated to Shandong First Medical University, Jinan, Shandong, China; b Department of Pathogen Biology, School of Basic Medicine, Shandong First Medical University and Shandong Academy of Medical Sciences, Jinan, China; c Key Lab for Biotech-Drugs of National Health Commission, Jinan, Shandong, China; d Key Lab for Rare and Uncommon Diseases of Shandong Province, Jinan, Shandong, China; e Institute of Clinical Microbiology, Shandong Academy of Clinical Medicine, Jinan, Shandong, China; University of Hong Kong

**Keywords:** *Salmonella* Typhimurium, regulation of flagellum synthesis, STM1344, STM1697, YdiV

## Abstract

When Salmonella enters host cells, the synthesis of flagella is quickly turned off to escape the host immune system. In this study, we investigated the cooperative regulatory mechanism of flagellar synthesis by two EAL-like proteins, STM1344 and STM1697, in Salmonella. We found that Salmonella upregulated the expression of both STM1344 and STM1697 to various degrees upon invading host cells. Importantly, deletion of STM1697 or STM1344 led to failure of Salmonella flagellar control within host cells, suggesting that the two factors are not redundant but indispensable. STM1697 was shown to modulate Salmonella flagellar biogenesis by preventing the flagellar master protein FlhDC from recruiting RNA polymerase. However, STM1344 was identified as a bifunctional factor that inhibits RNA polymerase recruitment of FlhDC at low molar concentrations and the DNA binding activity of FlhDC at high molar concentrations. Structural analysis demonstrated that STM1344-FlhD binds more tightly than STM1697-FlhD, and size exclusion chromatography (SEC) experiments showed that STM1344 could replace STM1697 in a STM1697-FlhDC complex. Our data suggest that STM1697 might be a temporary flagellar control factor upon Salmonella entry into the host cell, while STM1344 plays a more critical role in persistent flagellar control when Salmonella organisms survive and colonize host cells for a long period of time. Our study provides a more comprehensive understanding of the complex flagellar regulatory mechanism of Salmonella based on regulation at the protein level of FlhDC.

**IMPORTANCE** Salmonella infection kills more than 300,000 people every year. After infection, Salmonella mainly parasitizes host cells, as it prevents host cell pyroptosis by turning off the synthesis of flagellar antigen. Previous studies have determined that there are two EAL-like proteins, STM1344 and STM1697, encoded in the Salmonella genome, both of which inhibit flagellar synthesis by interacting with the flagellar master protein FlhDC. However, the expression order and simultaneous mechanism of STM1344 and STM1697 are not clear. In this study, we determined the expression profiles of the two proteins after Salmonella infection and demonstrated the cooperative mechanism of STM1344 and STM1697 interaction with FlhDC. We found that STM1344 might play a more lasting regulatory role than STM1697. Our results reveal a comprehensive flagellar control process after Salmonella entry into host cells.

## INTRODUCTION

Salmonella enterica serovar Typhimurium is an influential pathogen that causes food poisoning in humans and animals (1, 2). In a complex environment, Salmonella Typhimurium relies on flagella on the cell surface to avoid harm by moving (3). The presence of flagella is also closely related to pathogenic ability: before invasion, the flagella mediate the colonization, adhesion, and invasion process of Salmonella (4, 5); after invasion, flagellin molecules are recognized by the host Toll-like receptor 5 (TLR5), triggering the host immune response (6, 7). Wild Salmonella can rapidly shut down flagellar synthesis after entering host cells to achieve immune escape, while Salmonella that continuously expresses flagella will be rapidly recognized and killed by the host (8, 9). Therefore, the regulation of flagellar synthesis in Salmonella plays a critical role in pathogenesis.

The flagellum is the most massive extracellular organelle of Salmonella, and synthesis and assembly are highly ordered, hierarchical, and complex processes involving more than 200 genes on more than 70 operons (10–12). These genes are classified based on transcription order (13). The *flhDC* genes are transcribed early in the process and encode major regulator proteins, FlhD and FlhC (14, 15). Four FlhD proteins and two FlhC proteins assemble into a hexameric loop structure, the FlhD_4_C_2_ complex, which turns on flagellin expression by binding target genes and recruiting RNA polymerase to activate transcription (14, 16).

FlhD and FlhC are required to initiate the expression of all other flagellin proteins, and regulation of FlhDC expression limits flagellin synthesis (17, 18). Multiple regulatory factors regulate FlhD_4_C_2_ transcriptionally, posttranscriptionally, and translationally. At the transcriptional level, *flhDC* is regulated by cyclic AMP (cAMP)-cAMP receptor protein (CRP), H-NS (histone-like nucleoid structuring), Fur, RcsB, RtsB, and RflM (19–24). At the mRNA level, the RNA-binding protein CsrA upregulates flagellin expression by enhancing the translation of *flhDC* (25, 26). At the protein level, FlhD and FlhC are correctly folded into an active state with the help of the molecular chaperone DnaK to promote flagellar-gene expression, and the ClpXP protease complex recognizes and degrades FlhD_4_C_2_ to decrease flagellar-protein expression (16, 27). Three other factors, FliT, STM1344 (also known as YdiV), and STM1697, directly interact with FlhD_4_C_2_ (28–31). FliT acts on the FlhC subunit, and STM1344 and STM1697 interact with the FlhD subunit (31–33). In the case of limited genome capacity, Salmonella has evolved two proteins, STM1344 and STM1697, with highly similar functions, indicating the significance of the regulation process.

STM1344 and STM1697 share 29% sequence identity, and they belong to the same EAL family. The EAL family proteins, named for their characteristic EAL (Glu-Ala-Leu) motif, are typically associated with the hydrolysis of the second messenger cyclic di-GMP (c-di-GMP) (34). Sequence analysis revealed that both STM1344 and STM1697 proteins have mutations in key amino acids in the active center of the EAL structural domain. These proteins lack catalytic degradation activity and are unable to bind to c-di-GMP, but they are able to inhibit Salmonella mobility (35, 36).

Of these two Salmonella motility proteins that regulate flagellum synthesis by targeting the FlhD subunit, only one, YdiV (Salmonella STM1344 homolog), is present in Escherichia coli. We previously conducted an in-depth study of the mechanisms by which the E. coli YdiV and Salmonella STM1697 proteins regulate the flagellar pathway. We found that E. coli YdiV represses flagellar transcription by interfering with the DNA-binding activity of FlhD_4_C_2_, whereas Salmonella STM1697 represses flagellar transcription by reducing RNA polymerase recruitment (37, 38). However, it has not yet been determined if STM1344 in Salmonella functions like E. coli YdiV or like Salmonella STM1697. It is unclear why two EAL domain-like regulators are required for FlhD activity regulation.

In this study, we investigated the cooperative regulatory mechanism of flagellar synthesis by STM1344 and STM1697 in Salmonella. We found that Salmonella upregulated the expression of STM1344 and STM1697 upon invading host cells. However, the transcription level of STM1697 increased more than 100-fold within 2 h, while the transcription level of STM1344 increased more slowly at the beginning (10-fold in 2 h) and then rapidly increased more than 2,000-fold after 6 h postinfection, suggesting that STM1344 may play a more significant inhibitory role for the long-term survival of Salmonella within host cells. Global proteomic analysis showed that the deletion of STM1344 resulted in significantly increased levels of the monomeric subunit flagellin. Structural analysis and SEC experiments demonstrated that STM1344-FlhD binds more tightly than STM1697-FlhD. The regulation of the flagellar pathway by STM1344 is concentration dependent. At low molar concentration ratios (≤2:1 [STM1344 to FlhD_4_C_2_]), STM1344 acts similarly to STM1697, shutting down flagellin synthesis by inhibiting RNA polymerase recruitment of FlhD_4_C_2_. At high molar ratios (>2:1 [STM1344 to FlhD_4_C_2_]), STM1344 directly disrupts the FlhD4C2 hexameric ring structure, preventing FlhDC from binding to target genes; as a result, flagellar synthesis is turned off, and thus, Salmonella organisms escape from components of the immune system targeting flagellin. Our study provides a more comprehensive understanding of the complex flagellum-regulatory mechanism of Salmonella based on the regulation at the protein level of FlhDC.

## RESULTS

### STM1344 and STM1697 surge to a high level after Salmonella enters host cells.

**FIG 1:**
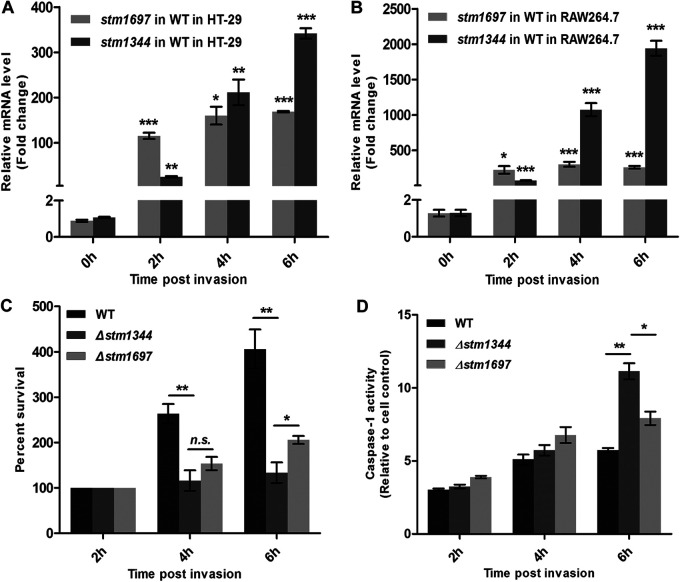
STM1344 might play a more critical role than STM1697 in the late phase of Salmonella infection. (A and B) The transcription levels of STM1344 and STM1697 in Salmonella before and after invasion of HT-29 and RAW264.7 cells were detected by qRT-PCR. *gapA* (glyceraldehyde-3-phosphate dehydrogenase A) was used as a loading control. (C) Intracellular survival capability of WT, Δ*stm1344*, and Δ*stm1697* strains in RAW264.7 cells at 2, 4, and 6 h postinfection. (D) Caspase-1 activity of RAW264.7 cells infected with the above strains after 2, 4, and 6 h postinfection. ***, *P* < 0.001; **, *P* < 0.01; *, *P* < 0.05.

We examined the transcriptional changes of STM1344 and STM1697 before and after invasion of HT-29 cells and RAW264.7 cells by Salmonella strain 14028 using quantitative reverse transcription-PCR (qRT-PCR). Compared with before invasion, STM1697 mRNA steady-state levels increased 129.7-, 179.9-, and 189.6-fold and STM1344 mRNA steady-state levels increased 23.3-, 197.6-, and 319.4-fold at 2, 4, and 6 h, respectively, after invasion of HT-29 cells (Fig. 1A). After invasion of RAW264.7 cells for 2, 4, and 6 h, STM1697 mRNA steady-state levels increased 175.3-, 237.5-, and 203.7-fold and STM1344 mRNA steady-state levels increased 58.6-, 832.9-, and 1,504.7-fold, respectively (Fig. 1B). Upon entering the host cells, STM1697 transcription starts rapidly and stabilizes at ~100- to 200-fold, while induction of STM1344 expression starts relatively slowly but eventually reaches several-hundred-fold or even several-thousand-fold. This high level of expression suggests that STM1344 may be more important for regulation in a more hostile environment.

### Both STM1344 and STM1697 play a critical role in flagellum control during Salmonella invasion.

**FIG 2:**
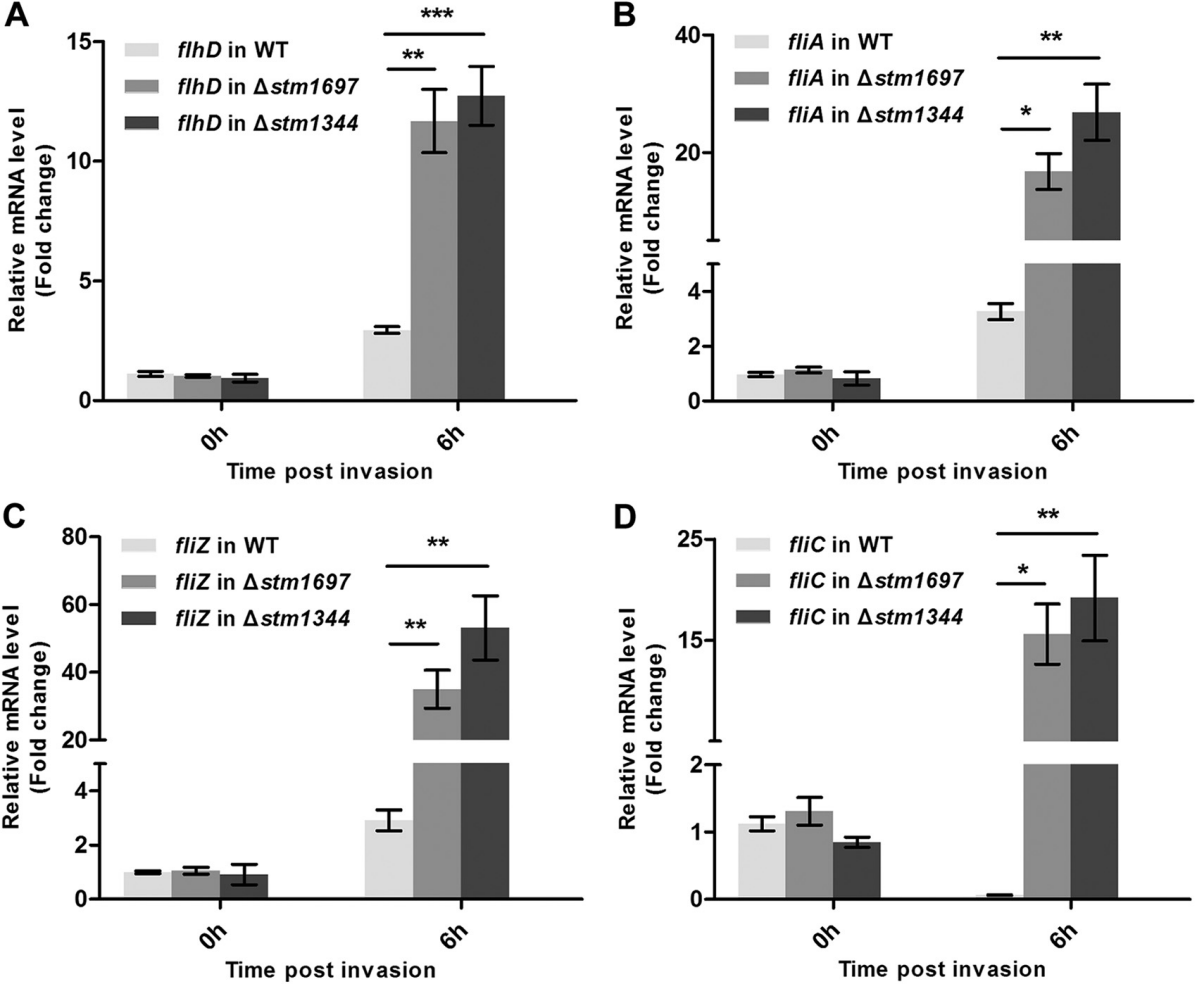
Both STM1344 and STM1697 are involved in flagellar control during Salmonella infection (A–D). The transcription levels of flagellar genes (*flhD*, *fliA*, *fliZ*, and *fliC*) in WT, Δ*stm1344*, and Δ*stm1697* strains before and after they entered RAW264.7 cells were detected by RT-qPCR. These experiments were performed as three replicates, and the mean values are presented. ***, *P* < 0.001; **, *P* < 0.01; *, *P* < 0.05.

To further investigate the function of *stm1344* and *stm1697* genes, we constructed single-gene-deletion (Δ*stm1344* and Δ*stm1697*) strains using Salmonella ATCC 14028 (wild type [WT]). Then, intracellular survival assays of these three bacterial strains in RAW264.7 cells were performed. The results showed that the survival rate of the Δ*stm1344* strain in RAW264.7 cells at 6 h postinfection was significantly lower than that of the WT and Δ*stm1697* strains (Fig. 1C), suggesting STM1344 works in the late stage of infection. In order to study the ability of bacteria to trigger pyroptosis of host cells, the caspase-1 activity of bacterium-infected RAW264.7 cells was detected. The results showed that the activity of caspase-1, the key protease in the pyrolytic signaling pathway, in Δ*stm1344* mutant-infected cells was significantly higher than that of WT- and Δ*stm1697* mutant-infected cells at 6 h postinfection (Fig. 1D). These results demonstrated that STM1344 did play a more critical role in flagellar control when Salmonella survived and colonized host cells for a long period of time. Because STM1344 and STM1697 were reported to be related to flagellar regulation, the transcription of flagellum-related genes before and after Salmonella entry into host cells was detected with the above-described three strains using qRT-PCR (Fig. 2A to D). The results showed significantly higher expression levels of flagellum-related gene transcripts in the Δ*stm1344* and Δ*stm1697* strains than in the WT strain, with higher levels in the Δ*stm1344* strain (260.17-fold *fliC* in the Δ*stm1697* strain and 319.67-fold *fliC* in the Δ*stm1344* strain). The results also suggest a potential role of STM1344 and STM1697 in transcription of class 1 *flhD* gene inside eukaryotic cells (Fig. 2A). These results support the importance of both STM1697 and STM1344 in the regulation of flagellum expression, while STM1344 may play a more important role in the long-term intracellular survival of Salmonella.

### STM1344 inhibits class II and III flagellar gene expression.

**FIG 3:**
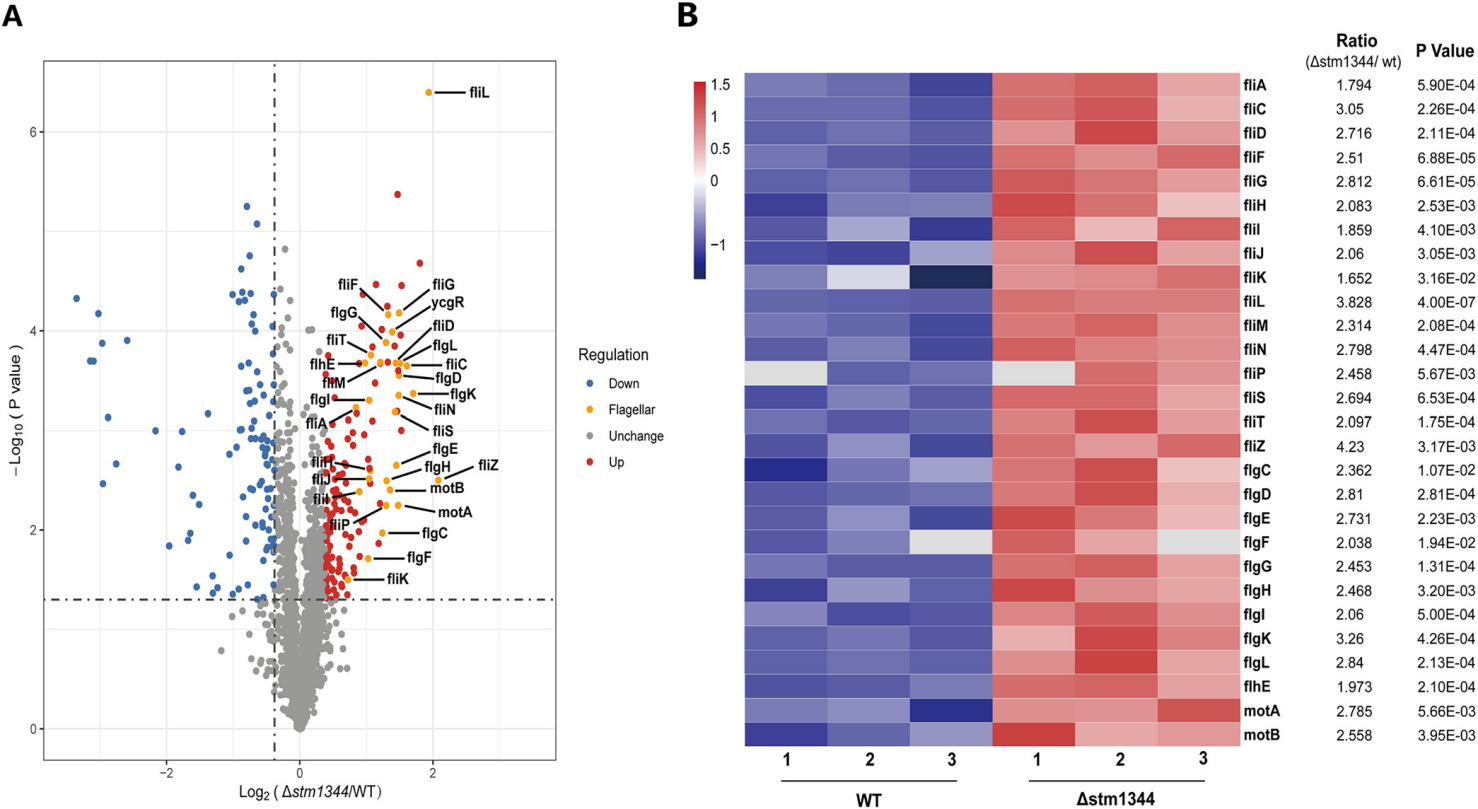
Flagellum-related proteins are significantly upregulated in the Δ*stm1344* mutant compared with the WT. The volcano plot (A) and heat map (B) show the expression difference of flagellar proteins in WT and Δ*stm1344* strains under host environmental pressure, determined by proteomic analysis. The proteins are arranged by classification in the heat map.

To further investigate the function of STM1344 to regulate flagellar-regulon protein expression after Salmonella entry into host cells, we cultured WT and Δ*stm1344* strains in medium simulating the host environment and analyzed the differences in protein expression between the two strains by mass spectrometry (MS)-based proteomics (Fig. 3; also, see Fig. S1 and S2 in the supplemental material). Under the simulated host environment stress, the expression levels of class 2 and 3 flagellar-regulon proteins were significantly higher in the Δ*stm1344* strain than the WT strain, with no significant differences detected in the expression of the primary flagellum-associated proteins FlhD and FlhC. This indicates that the regulation of flagellar synthesis by STM1344 occurred after the synthesis of FlhD and FlhC, reducing production of secondary and tertiary flagellar proteins. This is consistent with the previous finding that STM1344 regulates flagellar synthesis by acting as an anti-FlhDC factor.

### F181 and A184 of STM1344 are important for FlhD-binding.

**FIG 4:**
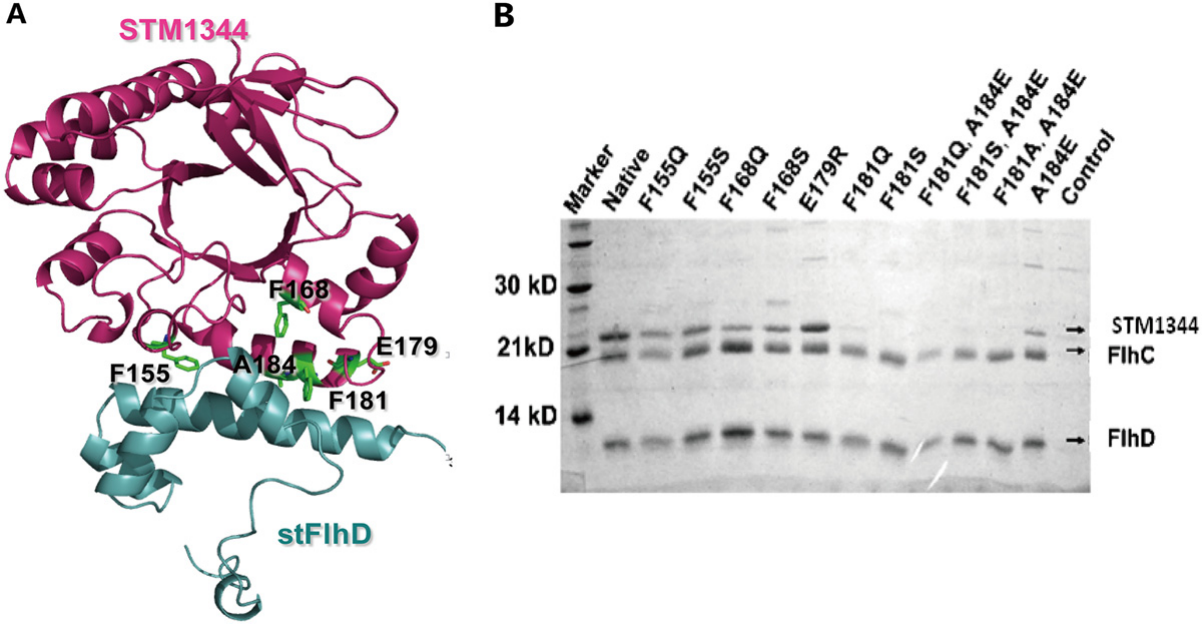
F181 and A184 of STM1344 are important for FlhD binding. (A) Interface between STM1344 and FlhD. STM1344 residues in the interface are shown as a stick model. (B) Pulldown of native and mutant STM1344 by FlhD_4_C_2_ with the His tag. The two mutated residues (F181 and A184) of STM1344 lose interaction with FlhD_4_C_2_.

The STM1344-FlhD complex was obtained by coexpression in E. coli BL21(DE3) and purification with a nickel-nitrilotriacetic acid (Ni-NTA) affinity column (Fig. S3). Using the crystal structure of the STM1344 homolog YdiV as a model, the structure of STM1344 was simulated and docked with an FlhD structure to obtain a structural model of the STM1344-FlhD complex (Fig. 4A). In the model, five amino acids (F155, F168, E179, F181, and A184) of STM1344 are predicted to interact with FlhD. These residues were selected for single or combined targeted mutation, and 11 mutant proteins were expressed or purified. Pulldown experiments demonstrated that F181 and A184 are key sites for STM1344 to bind to FlhD_4_C_2_, as the combined mutation of these two sites (F181Q+A184E, F181S+A184E, or F181A+A184E) results in the inability of STM1344 to bind to the FlhD_4_C_2_ complex (Fig. 4B).

### STM1344 inhibits flagellar synthesis with two different mechanisms.

**FIG 5:**
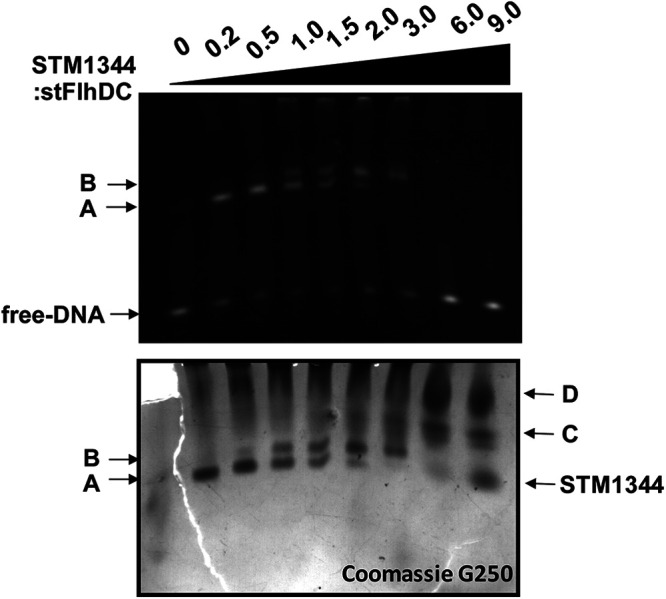
STM1344 forms different complexes with FlhD_4_C_2_. EMSA of FlhD_4_C_2_ and its target DNA at different concentration of STM1344. Band A is the complex of STM1344_1_-FlhD_4_C_2_ and DNA, band B is the complex of STM1344_2_-FlhD_4_C_2_ and DNA, band C is STM1344_3_-FlhD_4_C_2_, and band D is STM1344_4_-FlhD_4_C_2_.

We previously showed that low molecular ratio of STM1344 (STM1344 to FlhD_4_C_2_, ≤2:1, forming the STM1344_1_-FlhD_4_C_2_ and STM1344_2_-FlhD_4_C_2_ complexes) can affect the recruitment of RNA polymerase by FlhD_4_C_2_ (17), thereby shutting down transcription of downstream genes. Here, the results of electrophoretic mobility shift assays (EMSA) showed that high concentrations of STM1344 (STM1344 to FlhD_4_C_2_, >2:1, forming the STM1344_3_-FlhD_4_C_2_ and STM1344_4_-FlhD_4_C_2_ complexes) disrupted the binding of the FlhD_4_C_2_-DNA complex, blocking transcription of downstream flagellar genes (Fig. 5). These experimental results are consistent with previous research (33).

### STM1344 disrupts the structure of the STM1697-FlhD_4_C_2_ complex.

**FIG 6:**
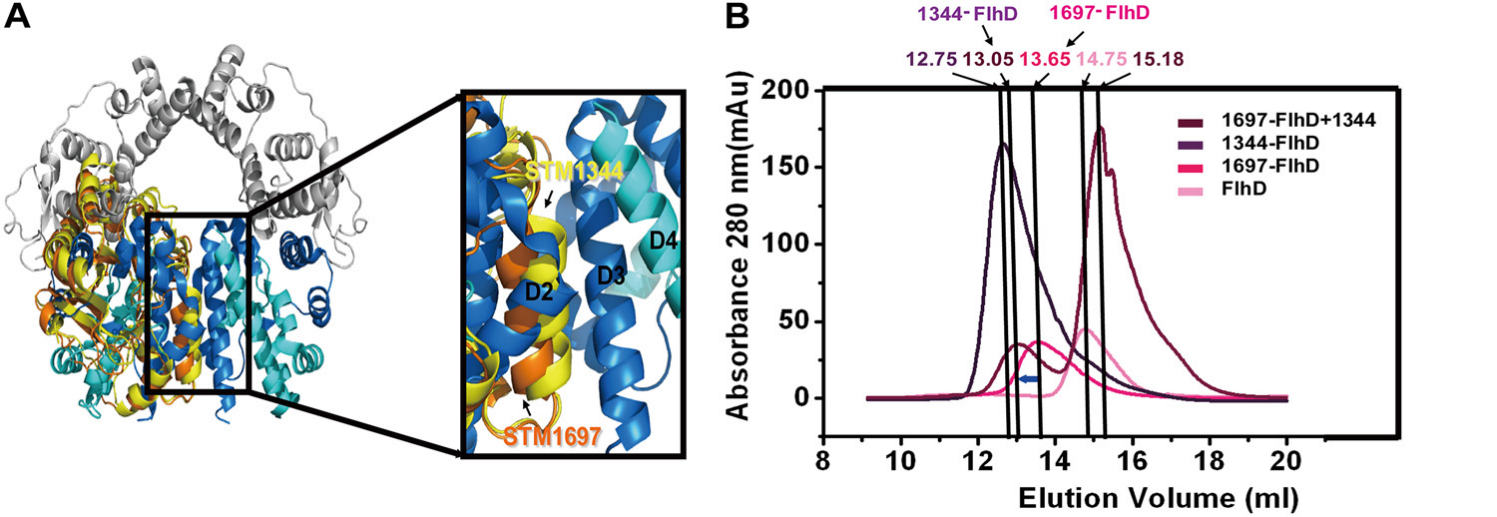
STM1344 disrupts the complex of the STM1697-FlhD_4_C_2_. (A) The structure of FlhD_4_C_2_ was used as the model, and STM1344-FlhD and STM1697-FlhD were separately superimposed onto the third FlhD molecule of FlhD_4_C_2_ (yellow, STM1344; orange, STM1697; blue, FlhD). The structures are shown in cartoon mode. (B) SEC assays showed that the involvement of STM1344 leads to the movement of the peak position from 13.65 mL to 13.05 mL, which is close to that of STM1344-FlhD (12.75 mL), suggesting that STM1344 was able to disrupt the STM1697-FlhD complex to form the STM1344-FlhD complex.

To explore which one has priority for combining with the FlhD_4_C_2_ complex when STM1344 and STM1697 are present at the same time, structure fitting comparison and size exclusion chromatography (SEC) experiments were performed. Structure fitting comparison revealed that the binding surface of STM1344 is closer to that of FlhD than that of STM1697 is (Fig. 6A), suggesting that the binding of STM1344 to FlhD may be tighter. Then, SEC assays were used to prove the above hypothesis. The SEC results showed that the involvement of STM1344 leads to the movement of the peak position from 13.65 mL to 13.05 mL, which is close to that of STM1344-FlhD (12.75 mL), suggesting that STM1344 is able to disrupt the STM1697-FlhD complex and instead form the STM1344-FlhD complex (Fig. 6B).

### STM1344 shuts down the flagellar synthesis pathway after Salmonella entry into cells to achieve immune escape and enhance pathogenicity.

**FIG 7:**
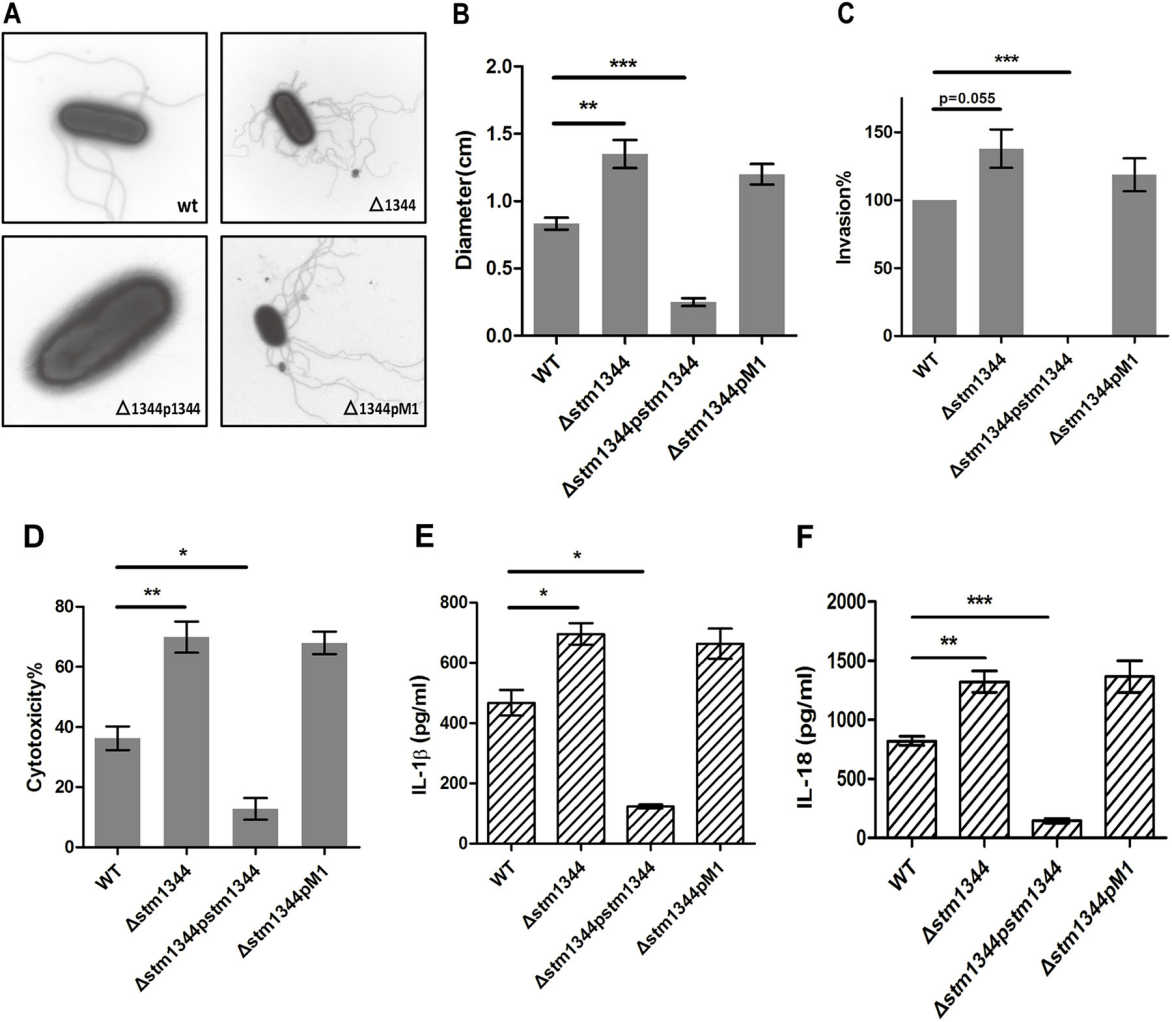
STM1344 regulates Salmonella motility and the immune response of host cells. (A) Electron micrographs of WT, Δ*stm1344*, Δ*stm1344*p*stm1344*, and Δ*stm1344*pM1 strains. (B) The motilities of the four strains described above were measured on a 0.3% LB agar plate. (C) Invasion capability of the four strains for HT-29 cells. (D) BMDM from BALB/c mice were infected with the four strains. At 4 h postinfection, cytotoxicity was detected by LDH release. (E and F) IL-1β and IL-18 secretion of BMDM infected with the four strains was detected by ELISA after 4 h postinfection. ***, *P* < 0.001; **, *P* < 0.01; *, *P* < 0.05.

Strains in which *stm1344* was deleted, overexpressed, or mutated in its key sites of FlhD interaction (F181A and A184E) were constructed using the wild-type Salmonella strain. The results of electron microscopy and swimming motility assays showed that STM1344 inhibits the motility of Salmonella by shutting down the flagellar synthesis pathway by interacting with the FlhD subunit, and the key sites for the interaction are F181 and A184 (Fig. 7A and B).

The above results show that STM1344 interacts with the FlhD subunit. We next investigated Salmonella virulence. Compared to the WT strains, invasion rates were significantly higher for Δ*stm1344* strains and back-complemented *stm1344* strains with the mutant strain (STM1344 mutated at this locus cannot bind to FlhD) and significantly lower for STM1344-overexpressing strains (Fig. 7C). Thus, STM1344 affects the rate of HT-29 cell invasion, suggesting that binding to FlhD is required. We next determined the ability of each strain to cause cell membrane damage upon macrophage infestation, i.e., macrophage toxicity, by measuring release of lactate dehydrogenase (LDH) and the cell death-related factors interleukin 1β (IL-1β) and IL-18 by ELISA. The release of LDH, IL-1β, and IL-18 was elevated in the Δ*stm1344* strain and the back-complemented *stm1344* strain with a point mutation compared with the WT strain, with significantly lower release in the overexpression strain (Fig. 7D to F). This result indicated that STM1344 significantly inhibits Salmonella-induced macrophage pyroptosis and that this effect requires interaction with the FlhD subunit.

**FIG 8:**
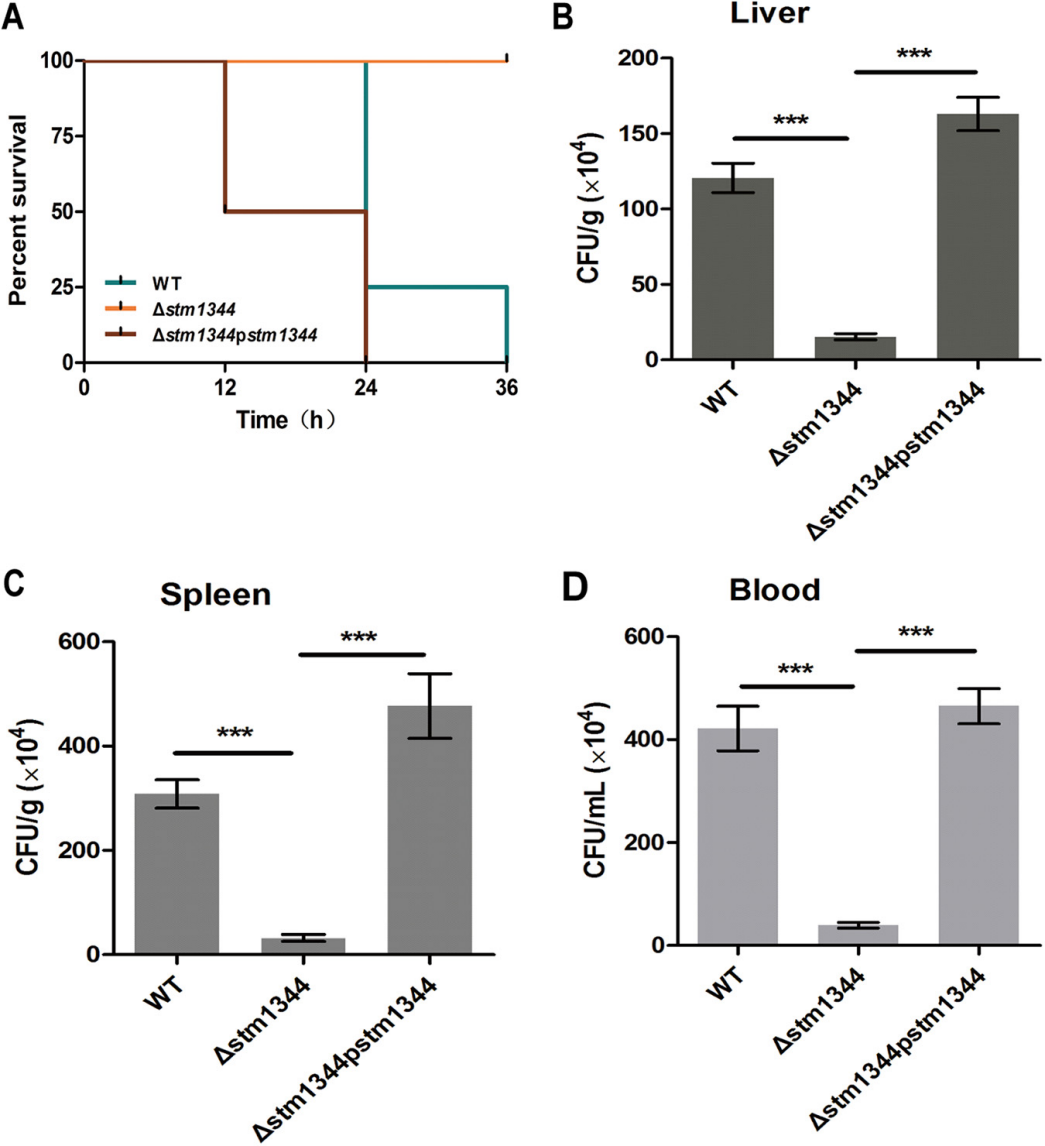
STM1344 modulates the pathogenicity of Salmonella infected-animals. (A) BALB/c mice were infected with equal numbers of WT, Δ*stm1344*, and Δ*stm1344*p*stm1344* strains by intraperitoneal injection. Survival was observed every 2 to 3 h. (B to D) Numbers of Salmonella organisms in liver, spleen, and blood of BALB/c mice infected with the three strains. ***, *P* < 0.001; **, *P* < 0.01; *, *P* < 0.05.

To verify the effect of STM1344 on the pathogenicity of Salmonella *in vivo*, we performed mouse experiments. BALB/c mice were infected intraperitoneally with equal numbers (1 × 10^6^) of WT, Δ*stm1344*, and Δ*stm1344*p*stm1344* strains. For each strain, animal survival curves and bacterial loads in infected mice were determined. The mortality of mice infected with the Δ*stm1344* mutant was remarkably lower than that of mice infected with the WT and Δ*stm1344*p*stm1344* strains (Fig. 8A). The bacterial loads in the liver, spleen, and blood of the Δ*stm1344* strain-infected mice were significantly lower than those of WT-infected mice (Fig. 8B to D). These results suggested that STM1344 plays an important role in Salmonella infection by shutting down flagellar synthesis and achieving immune escape.

## DISCUSSION

The flagellum provides essential motility in the pathogenic process of Salmonella host invasion (39). However, flagellin is strongly antigenic and can be recognized by TLR5, resulting in activation of the host immune system to clear the invading pathogen (40, 41). The presence of flagella also disrupts the integrity of the bacterial cytosol, making it more vulnerable to external adversities (42). Salmonella must rapidly shut down the flagellar synthesis system after entering the host cell to evade the host immune system and resist damage from external adverse factors (9, 43). Both STM1344 and STM1697 proteins target FlhD protein and regulate this process. However, there was no previous comprehensive explanation of the characteristics and mechanisms of action of these two proteins, which act at the same site and perform the same function.

Our previous study showed that STM1697 affects the recruitment of RNA polymerase by binding to the outer two FlhD subunits of the FlhD_4_C_2_ complex, temporarily inhibiting the transcription of downstream flagellar genes for regulation of flagellar synthesis (38). In this study, we showed that, unlike STM1697, STM1344 exhibits concentration-dependent regulation of the flagellar pathway. At low molar concentrations (STM1344-FlhD_4_C_2_ ratio, ≤2:1), STM1344 takes advantage of its stronger binding to FlhD to disrupt the binding of STM1697 to FlhD, forming STM1344_1_-FlhD_4_C_2_ and STM1344_2_-FlhD_4_C_2_ complexes. These complexes inhibit the recruitment of FlhD_4_C_2_ to RNA polymerase and shut down transcription of downstream flagellar genes. At high molar concentrations (STM1344-FlhD_4_C_2_ ratio, >2:1), STM1344 can also bind to the two FlhD subunits on the inner side of the FlhD_4_C_2_ complex to form STM1344_3_-FlhD_4_C_2_ and STM1344_4_-FlhD_4_C_2_ complexes. which directly disrupt the FlhD_4_C_2_ hexameric ring structure. This disruption of the FlhD_4_C_2_ hexameric loop structure leads to the loss of the ability to bind to target DNA and regulate transcription. It has also been reported that STM1344 promotes the degradation of the FlhD_4_C_2_ complex by ClpXP protease, for additional negative regulation of bacterial flagellar synthesis (30).

We found that STM1344 and STM1697 play different roles at different times during the regulation of flagellar synthesis after Salmonella invasion of host cells. Before Salmonella entry into host cells, the expression levels of STM1344 and STM1697 were low, and the FlhD_4_C_2_ complex bound to the region upstream of the downstream flagellum gene and recruited RNA polymerase to initiate transcription of the flagellum gene. Upon entering the host cell, Salmonella senses an unknown signal as the external environment changes to respond rapidly to evade host recognition and killing.

First, the transcriptional expression of STM1697 is rapidly increased 100-fold (within 2 h), and because of the binding of STM1697 to two FlhD subunits on the outer side of the FlhD_4_C_2_ complex, the FlhD_4_C_2_-DNA complex cannot recruit RNA polymerase. This rapidly turns off the transcriptional expression of downstream genes of the flagellum as an early, temporary, and reversible regulation of flagellum synthesis. If the stimulatory signal persists, Salmonella turns on the transcriptional expression of STM1344. Although the transcriptional expression of STM1344 starts relatively slowly, it can increase several-hundred-fold (HT-29 cells) or even several-thousand-fold (RAW264.7 cells) in 6 h. At lower levels, STM1344 substituted for STM1697 to bind to the outer two FlhD subunits of the FlhD_4_C_2_ complex and hindered the recruitment of RNA polymerase to the FlhD_4_C_2_-DNA complex. At higher concentrations, STM1344 directly disrupted the hexameric ring structure of FlhD_4_C_2_, leading to complete disassembly of the transcriptional complex of genes downstream of the flagellum and directing the hydrolysis of the FlhD_4_C_2_ complex by the ClpXP protease (30), exerting a more prolonged and complete shutdown of flagellar synthesis.

**FIG 9:**
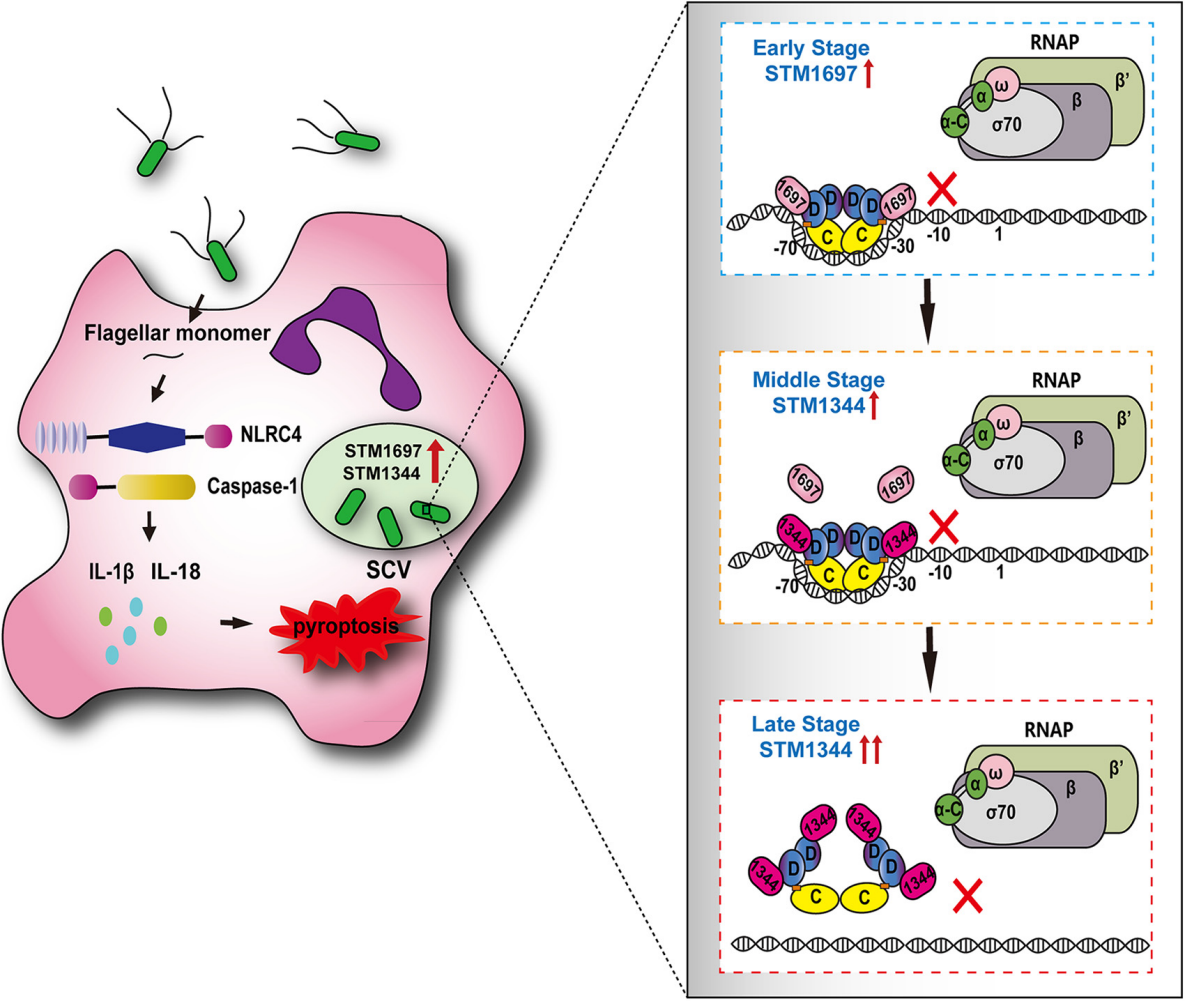
Cooperative regulatory mechanism of flagellar synthesis by STM1344 and STM1697 in Salmonella. When Salmonella enters host cells, the expression of both STM1344 and STM1697 is upregulated to various degrees and the synthesis of flagellum is quickly turned off to escape the host immune system. In this study, we investigated the idea that STM1697 might be a temporary flagellar control factor acting on Salmonella entry into host cells by preventing the flagellar master protein FlhDC from recruiting RNA polymerase. STM1344 plays a more critical role in persistent flagellar control when Salmonella survives and colonizes host cells for a long period of time by replacing STM1697 in a STM1697-FlhDC complex and removing the DNA binding activity of FlhDC at high molar concentrations. Our study provides a more comprehensive understanding of the complex flagellar regulatory mechanism of Salmonella based on the regulation of the protein level of FlhDC.

When Salmonella variants lacking STM1344 or STM1697 were allowed to invade RAW264.7 cells, the transcript levels of both secondary and tertiary flagellin genes were significantly increased after 6 h compared with the levels in the WT strain, with greater effects seen for the strain lacking STM1344. This result suggested that STM1344 plays a critical role in the regulation of late and persistent flagellar synthesis after host cell entry. As an intracellular parasite, Salmonella enters the host cell and, under the joint action of these two proteins, rapidly and persistently reduces the expression level of flagellin, thus effectively avoiding clearance by the host immune system and achieving immune escape (Fig. 9).

Salmonella expresses only low levels of STM1344 and STM1697 when cultured in nutrient-rich medium. However, after host cell invasion, the transcription level of STM1697 gene is upregulated hundreds of times in just 2 h. The transcription of the STM1344 gene, although relatively low initially, increases by several-hundred-fold or even 2,000-fold in 6 h. During host invasion, Salmonella is exposed to dramatically different conditions with regard to pH, reactive oxygen concentration, metal ion concentration, and nutrient status. Future work should investigate the stimuli that prompt Salmonella to activate the expression of STM1344 and STM1697 and the specific mechanisms of activation.

## MATERIALS AND METHODS

### Bacterial strains and culture media.

The Salmonella Typhimurium strain used was ATCC 14028 (WT). The *stm1344* and *stm1697* knockout (Δ*stm1344* and Δ*stm1697*) mutants of ATCC 14028 were constructed using the λRed recombinase system as described previously (44). Luria broth (LB) or LB agar plates were prepared as described previously (45). The simulated host environment medium was iron-deficient medium supplemented with 600 μM bipyridine. Motility agar plates were prepared as described previously (45).

### Plasmid construction and protein expression and purification.

The STM1344, STM1697, and *S.* Typhimurium FlhD (StFlhD) genes were cloned into the prokaryotic expression vector pGL01, and the entire *flhDC* operon was cloned into pET21b. The StFlhD gene was cloned into pET29b, which does not contain the His tag. The mutant plasmids of STM1344 were constructed based on the plasmid STM1344-pGL01 using a site-directed mutagenesis system (TransGen Biotech, Beijing, China). For *in vivo* experiments, the STM1344 and STM1344-M1 (F181A and A184E) genes were cloned into the pBAD24 vector. STM1344 and 11 mutants were expressed in E. coli BL21(DE3) using LB medium with isopropyl-β-d-thiogalactopyranoside (IPTG) and purified with a Ni^2+^-NTA affinity column and Superdex 200. The STM1344-StFlhD complex was coexpressed in E. coli BL21(DE3) using LB medium as described previously (37). Then, the complex was lysed with trypsin and purified using a Source Q ion exchange column and Superdex 200 chromatography. StFlhD_4_C_2_ was expressed and purified as described previously (21).

### Proteomic analysis.

Tandem mass tag (TMT) quantitative proteomic analysis was performed by Jingjie Biological Technology Co., Ltd. (Hangzhou, China). Briefly, total protein was extracted from WT and Δ*stm1344* strains using a high-intensity ultrasonic processor (Scientz) in lysis buffer. The supernatant was collected by centrifugation and quantified protein concentration with a bicinchoninic acid (BCA) assay kit according to the manufacturer’s instructions. After trypsin digestion, the peptide was desalted with a Strata X-C18-SPE column (Phenomenex) and processed according to the manufacturer’s protocol for the TMT kit/iTRAQ kit. Then, the tryptic peptides were fractionated by high-pH (pH 9.0) reverse-phase high-performance liquid chromatography (HPLC) using an Agilent 300Extend C_18_ column. After liquid chromatography-tandem mass spectrometry (LC-MS/MS) analysis, the data were processed using the Maxquant search engine (v.1.5.2.8).

### Pulldown assay.

FlhD_4_C_2_ with a His tag was mixed with HisLink protein purification resin (Promega, Madison, WI) in the presence or absence of STM1344 and mutants without the His tag. After gentle shaking at 4°C for 2 h, the resin was collected by centrifugation, washed five times with 1 mL of wash buffer (50 mM NaH_2_PO_4_, 300 mM NaCl, 20 mM imidazole [pH 8.0]), and resuspended in elution buffer (50 mM NaH_2_PO_4_, 300 mM NaCl, 250 mM imidazole [pH 8.0]). After centrifugation, proteins in the supernatant were separated by SDS-PAGE.

### EMSA experiment.

A 49-bp *flhB* promoter of Salmonella was synthesized as the target DNA. The target DNA was preincubated with different ratios of proteins for 10 min. Then the mixtures were separated on a native 5% polyacrylamide gel and stained with ethidium bromide and Coomassie brilliant blue.

### Size exclusion chromatography.

FlhD was mixed at the appropriate ratio with STM1344, STM1697, and STM1344 plus STM1697. After incubation for 10 min at 4°C, the mixtures were injected for SEC using a Superdex 200 column.

### Cell culture and preparation of bacteria.

HT-29 (human colon adenocarcinoma) cells were cultured at 37°C in a humidified atmosphere with 5% CO_2_ in complete RPMI 1640 medium (Gibco) containing 10% fetal bovine serum (Gibco). RAW264.7 (mouse mononuclear macrophage leukemia cells), and bone marrow-derived macrophages (BMDM) were cultured under the same conditions in Dulbecco’s modified Eagle medium (DMEM) (Gibco) containing 10% fetal bovine serum (Gibco). Salmonella strains were grown overnight in 10 mL LB medium with 100 μg/mL ampicillin and then transferred into 5 mL LB medium plus 0.3 M NaCl to induce the invasion phenotype with 0.1% l-arabinose, in order to induce protein expression. All strains were harvested at an optical density at 600 nm (OD_600_) of 0.4 to 0.6 and adjusted to the same concentration.

### Transmission electron microscopy.

Salmonella strains were cultured in LB medium with IPTG, harvested in logarithmic phase, and observed by transmission electron microscopy.

### Swimming motility assay.

Bacteria were diluted to an OD_600_ of 10 and inoculated on 0.3% LB agar plates with 100 μg*/*mL ampicillin and 0.1% l-arabinose. Swimming motility was measured after 5 h at 30°C.

### Invasion assay.

Salmonella strains were seeded on HT-29 cell monolayers grown in 96-well plates at a multiplicity of infection (MOI) of 20. One hour postinfection, gentamicin (100 μg/mL) was added to the cells for 1 h to kill the remaining extracellular bacteria. Then cells were washed gently with phosphate-buffered saline and lysed with 1% Triton X-100 (Sigma Chemical). The number of intracellular bacteria was detected by the CFU counts of viable colonies.

### Macrophage cytotoxicity assay.

Bone marrow-derived macrophages were infected with Salmonella strains at an MOI of 10 and then incubated for 4 h. Cytotoxicity was detected by an LDH assay (CytoTox 96; Promega). The concentrations of IL-1β and IL-18 in the supernatants were determined with an enzyme-linked immunosorbent assay (ELISA) kit (R&D Systems and MBL, respectively).

### Bacterial infection model.

HT-29 and RAW264.7 were seeded at 1 × 10^7^ in 100-mm-diameter tissue culture dishes. Salmonella strains were added to the cells at an MOI of 10. After 1h at 37°C, 100 μg/mL gentamicin was added to the cells to kill the extracellular bacteria. At each time point postinfection (2, 4, and 6 h), the cells were washed with NS, lysed in TRIzol reagent (Tiangen), and stored at −70°C.

### RNA extraction and real-time RT-qPCR.

Total RNA was extracted according to the TRIzol reagent manufacturer’s instructions. Reverse transcription reactions were performed with the RevertAid cDNA synthesis kit (Thermo). qRT-PCR was performed with an Applied Biosystems 7500 sequence detection system (Applied Biosystems, Foster, CA, USA) with iTaq universal SYBR green supermix (Bio-Rad).

### Intracellular survival assay.

RAW264.7 cells were seeded at 1 × 10^5^ in 96-well plates. Salmonella strains were added to the cells at an MOI of 10. After 1 h at 37°C, 100 μg/mL gentamicin was added to the cells to kill the extracellular bacteria. At each time point postinfection (2, 4, and 6 h), the cells were washed with phosphate-buffered saline and lysed with 1% Triton X-100 (Sigma Chemical). The number of intracellular bacteria was determined by the CFU counts of viable colonies.

### Caspase-1 activity assay.

RAW264.7 cells were seeded at 2 × 10^6^ in 6-well plates. Salmonella strains were added to the cells at an MOI of 20. After 1 h at 37°C, 100 μg/mL gentamicin was added to the cells to kill the extracellular bacteria. At each time point postinfection (2, 4, and 6 h), the cells were harvested, and caspase-1 activity was determined with assay kits (Beyotime Biotechnology).

### Animal experiment.

All animal procedures were approved by the Ethics Committee of Animal Care and Use, Institute of Basic Medicine, Shandong Academy of Medical Sciences. In animal experiments, 4-week-old female BALB*/*c mice were used. For survival study, each group of mice (*n *= 8) was infected intraperitoneally with 1 × 10^6^ CFU of each *S.* Typhimurium strain, and the survival of mice were observed every 2 to 3 h. For serum test, each group of mice (*n *= 6) was infected with each *S.* Typhimurium strain in the same scheme. At each time point postinfection (6 and 12 h), serum was obtained from 3 mice in each group. For bacterial counts, mice (*n *= 6) were infected with 1 × 10^6^ CFU of each S. Typhimurium strain. At 6 h postinfection, the mice were sacrificed and the numbers of bacteria in the spleen, liver, and blood were detected by CFU counts of viable colonies.

### Data availability.

Proteomic data have been submitted to ProteomeXchange via the PRIDE database (http://www.ebi.ac.uk/pride) under the data set identifier PXD035693.
