# Supplementary material for: Cooperative Regulation of Flagellar Synthesis by Two EAL-Like Proteins upon Salmonella Entry into Host Cells
Source: Microbiol Spectr. 2023 Feb 7;11(2):e02859-22. doi: 10.1128/spectrum.02859-22 (PMC10100727; doi:10.1128/spectrum.02859-22)
Supplement: Supplemental file 1 — Supplemental material. Download spectrum.02859-22-s0001.pdf, PDF file, 0.3 MB [file spectrum.02859-22-s0001.pdf]

SUPPLEMENTAL MATERIALS

Cooperative Regulation of Flagellar Synthesis by Two EAL-like Proteins upon *Salmonella* Entry into Host Cell

Yingying Yue[a,b*], Weiwei Wang[a,b], Yue Ma[a,b], Nannan Song[a,b], Haihong Jia[a,b], Cuiling Li[a,b], Qi Wang[a,b], Hui Li[a,b], and Bingqing Li[a,b,c,d,e*]

[a] Department of Clinical Laboratory, Shandong Provincial Hospital Affiliated to Shandong First Medical University, Jinan, 250021, Shandong, China;

[b] Department of Pathogen Biology, School of Basic Medicine, Shandong First Medical University & Shandong Academy of Medical Sciences, Jinan, 250062, China.

[c] Key Lab for Biotech-Drugs of National Health Commission, Jinan 250062, Shandong, China;

[d] Key Lab for Rare & Uncommon Diseases of Shandong Province, Jinan, 250062, Shandong, China.

[e] Institute of clinical microbiology, Shandong Academy of Clinical Medicine, Jinan, Shandong, China.

*Correspondence: Address correspondence to Bingqing Li, bingqingsdu@163.com, Yingying Yue, yyy0513@163.com.

Figure S1-S3

Table S1 S2

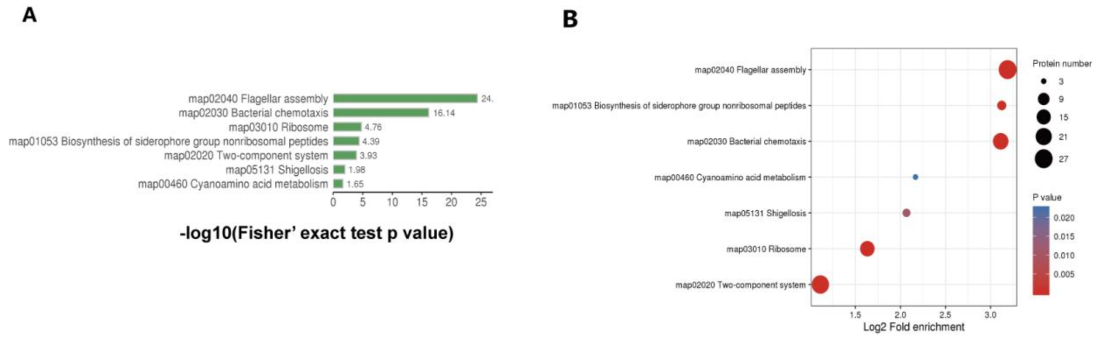

Figure S1. KEGG enrichment analysis of the WT *and* Δ*stm1344* strains pathways regulated in simulated host environment medium.

Related biological function of all 7 pathways are displayed in bar (A) and dot (B), and bar height or dot size indicates the level of significance of enrichment. The flagella assembly is most significant.

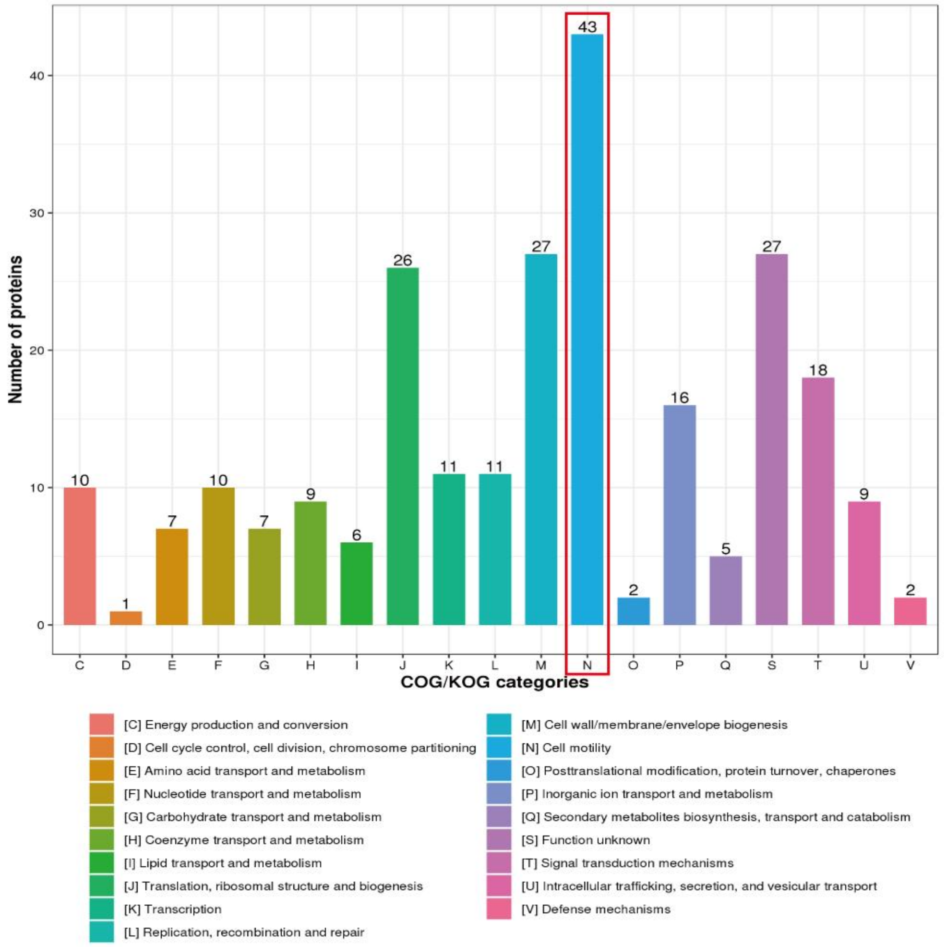

Figure S2. COG/KOG categories analysis of the WT *and* Δ*stm1344* strains in simulated host environment medium.

Protein number with different functions are displayed in bar. The difference of cell motility related proteins represents the greatest significant.

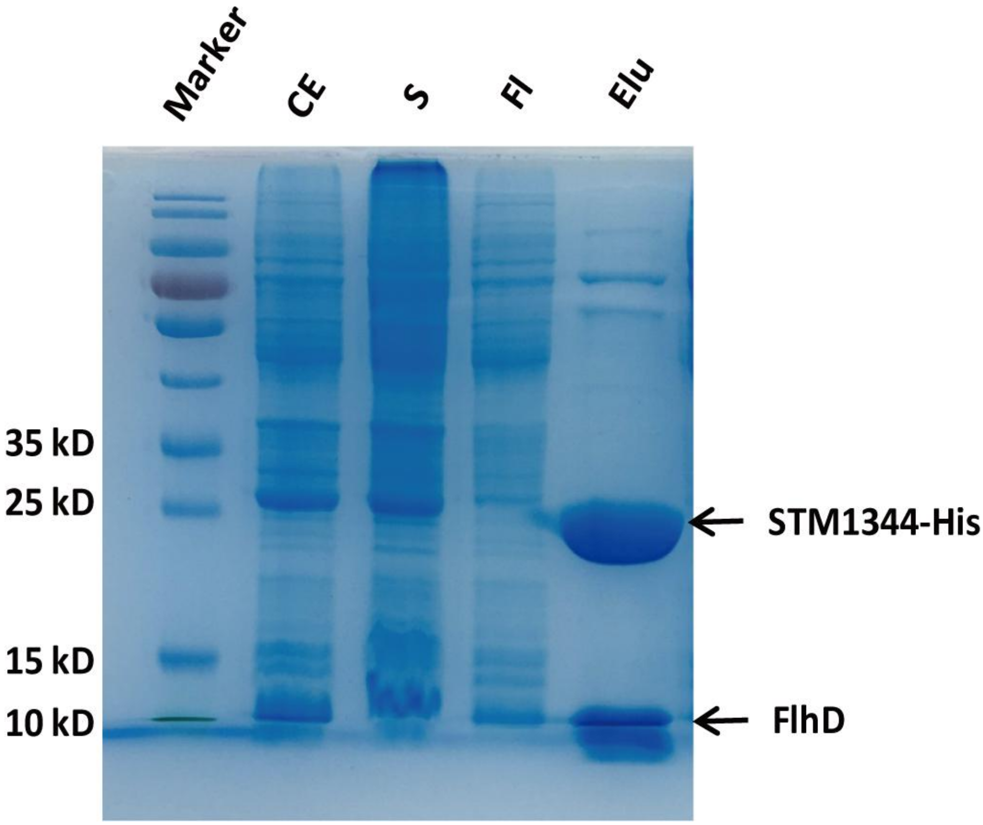

Figure S3. The expression and purification of STM1344-FlhD complex.

STM1344-FlhD complex samples were separated by SDS-PAGE and shown after coomassie blue staining. Lane Marker, protein marker; lane CE, the sample of cell lysate after induction; lane S, the supernatant of cell lysate after centrifugation; lane Fl, the sample flow through Ni-NTA affinity column; lane Elu, purified STM1344-FlhD complex.

Table S1. Strains and plasmids used in this study

No	Strain/Plasmid	Relevant characteristic(s)	Source
1	WT *Salmonella*	*Salmonella enterica serovar Typhimurium* ATCC14028, no resistance	American Type Culture Collection
2	*Salmonella* Δ*stm1344*	*stm1344* knockout strain, no resistance	This study
3	*Salmonella* Δ*stm1697*	*stm1697* knockout strain, no resistance	[1]
4	*Salmonella* Δ*stm1344pstm1344*	*stm1344* knockout, *stm1344*/pBAD24, Amp^+	This study
5	*Salmonella* Δ*stm1344p*M1	*stm1344* knockout, *stm1344* F181A & A184E/pBAD24, Amp^+	This study
6	*E. coli* BL21(DE3)	T7 expression host, no resistance	Takara Bio Inc.
7	pGL01	Expression Vector, Amp^+	[2]
8	pET29b	Expression Vector, $Kana^+$	Biofeng Inc.
9	pBAD24	Expression Vector, Amp^+	Biofeng Inc.
10	STM1344/pGL01	STM1344 cloned into pGL01	[1]
11	STM1697/pGL01	STM1697 cloned into pGL01	[1]
12	stFlhD/pGL01	stFlhD cloned into pGL01	[1]
13	stFlhD/pET29b	stFlhD cloned into pET29b	[1]
14	STM1344/pBAD24	STM1344 cloned into pBAD24	This study
15	STM1344 F181A&A184E /pBAD24	STM1344 F181A&A184E mutant protein expression construct into pBAD24	This study
16	STM1344 F155Q/pGL01	STM1344 F155Q mutant protein expression construct into pGL01	This study
17	STM1344 F155S/pGL01	STM1344 F155S mutant protein expression construct into pGL01	This study
18	STM1344 F168Q/pGL01	STM1344 F168Q mutant protein expression construct into pGL01	This study
19	STM1344 F168S/pGL01	STM1344 F168S mutant protein expression construct into pGL01	This study
20	STM1344 E179R/pGL01	STM1344 E179R mutant protein expression construct into pGL01	This study
21	STM1344 F181Q/pGL01	STM1344 F181Q mutant protein expression construct into pGL01	This study
22	STM1344 F181S/pGL01	STM1344 F181S mutant protein expression construct into pGL01	This study
23	STM1344 A184E/pGL01	STM1344 A184E mutant protein expression construct into pGL01	This study
24	STM1344 F181Q&A184E /pGL01	STM1344 F181Q&A184E mutant protein expression construct into pGL01	This study
25	STM1344 F181S&A184E / pGL01	STM1344 F181S&A184E mutant protein expression construct into pGL01	This study
26	STM1344 F181A&A184E / pGL01	STM1344 F181A&A184E mutant protein expression construct into pGL01	This study

[1] Li B, Yue Y, Yuan Z, Zhang F, Li P, Song N, Lin W, Liu Y, Yang Y, Li Z, Gu L. 2017. *Salmonella* STM1697 coordinates flagella biogenesis and virulence by restricting flagellar master protein FlhD4C2 from recruiting RNA polymerase. Nucleic Acids Research 45:9976-9989.

[2] Li B, Li N, Wang F, Guo L, Huang Y, Liu X, Wei T, Zhu D, Liu C, Pan H, Xu S, Wang H-W, Gu L. 2012. Structural insight of a concentration-dependent mechanism by which YdiV inhibits *Escherichia coli* flagellum biogenesis and motility. Nucleic Acids Research 40:11073-11085.

Table S2. Primers used in this study

No	Oligonucleotide	Sequence	Application
1	gapa-5	gaccttcgatgatgccgaag	qPCR
2	gapa-3	gccaggacatcgtttccaac	qPCR
3	stm1344-5	cagcagcgagctgaaatgatc	qPCR
4	stm1344-3	cgcaaacatcgcctcagtatc	qPCR
5	stm1697-5	ccaccacgatcttgtcac	qPCR
6	stm1697-3	ctgctgcgccattagtttgc	qPCR
7	flhD-5	cgcctcggtatcaacgaaga	qPCR
8	flhD-3	ctccgccagtttgaccatct	qPCR
9	fliA-5	cttacccagtttggtgcg	qPCR
10	fliA-3	cgagcaactggtgttaac	qPCR
11	fliZ-5	cagaactggcggtaaaggg	qPCR
12	fliZ-3	catttcccacgatctgctgc	qPCR
13	fliC-5	cgcagtaaagagaggacg	qPCR
14	fliC-3	gggcaacaccgtaaacaacc	qPCR
15	STM1344-pGl01-F	ATAGGATCCatgattgcttcacttgatgagc	Gene Clone
16	STM1344-pGl01-R	ATACTCGAGTTAtcgctgaacgagtttaatgagc	Gene Clone
17	STM1344-pBAD24-F	ATAGAATTCatgattgcttcacttgatg	Gene Clone
18	STM1344-pBAD24-R	AAGCTTAAGCTTTTAaacgagtttaatgagctggc	Gene Clone
19	STM1344-F155Q-F	acaaaagctattCAGgatgggcttttc	Gene Clone
20	STM1344-F155Q-R	gaaaagcccatcCTGaatagcttttgt	Gene Clone
21	STM1344-F155S-F	acaaaagctattAGCgatgggcttttc	Gene Clone
22	STM1344-F155S-R	gaaaagcccatcGCTaatagcttttgt	Gene Clone
23	STM1344-F168Q-F	ttggataaaaatCAGattcagcagcga	Gene Clone
24	STM1344-F168Q-R	tcgctgctgaatCTGatttttatccaa	Gene Clone
25	STM1344-F168S-F	ttggataaaaatAGCattcagcagcga	Gene Clone
26	STM1344-F168S-R	tcgctgctgaatGCTatttttatccaa	Gene Clone
27	STM1344-E179R-F	atgatctcattcCGAccattcatgcac	Gene Clone
28	STM1344-E179R-R	gtgcatgaatggTCGgaatgagatcat	Gene Clone
29	STM1344-F181Q-F	tcattcgaaccaCAGatgcacgctatc	Gene Clone
30	STM1344-F181Q-R	gatagcgtgcatCTGtggttcgaatga	Gene Clone
31	STM1344-F181S-F	tcattcgaaccaAGCatgcacgctatc	Gene Clone
32	STM1344-F181S-R	gatagcgtgcatGCTtggttcgaatga	Gene Clone
33	STM1344-A184E-F	tcattcgaaccaCAGatgcacGAAatcgttgcccag	Gene Clone
34	STM1344-A184E-R	ctgggcaacgatTTCgtg	Gene Clone
